# Hyperventilation Therapy for Control of Posttraumatic Intracranial Hypertension

**DOI:** 10.3389/fneur.2017.00250

**Published:** 2017-07-17

**Authors:** Daniel Agustín Godoy, Ali Seifi, David Garza, Santiago Lubillo-Montenegro, Francisco Murillo-Cabezas

**Affiliations:** ^1^Neurointensive Care Unit, Sanatorio Pasteur, San Fernando del Valle de Catamarca, Argentina; ^2^Intensive Care Unit, Hospital San Juan Bautista, Catamarca, Argentina; ^3^University of Texas Health Science Center San Antonio, San Antonio, TX, United States; ^4^Department of Neurosurgery, University of Texas Health Science Center San Antonio, San Antonio, TX, United States; ^5^Intensive Care Unit, Hospital Universitario N^a^ S^a^ de Candelaria, Santa Cruz de Tenerife, Spain; ^6^Intensive Care Unit, Hospital Universitario Virgen del Rocío, Sevilla, Spain

**Keywords:** hyperventilation, intracranial hypertension, intracranial pressure, hypocapnia, cerebral ischemia, cerebral hypoxia, severe traumatic brain injury

## Abstract

During traumatic brain injury, intracranial hypertension (ICH) can become a life-threatening condition if it is not managed quickly and adequately. Physicians use therapeutic hyperventilation to reduce elevated intracranial pressure (ICP) by manipulating autoregulatory functions connected to cerebrovascular CO_2_ reactivity. Inducing hypocapnia *via* hyperventilation reduces the partial pressure of arterial carbon dioxide (PaCO_2_), which incites vasoconstriction in the cerebral resistance arterioles. This constriction decrease cerebral blood flow, which reduces cerebral blood volume and, ultimately, decreases the patient’s ICP. The effects of therapeutic hyperventilation (HV) are transient, but the risks accompanying these changes in cerebral and systemic physiology must be carefully considered before the treatment can be deemed advisable. The most prominent criticism of this approach is the cited possibility of developing cerebral ischemia and tissue hypoxia. While it is true that certain measures, such as cerebral oxygenation monitoring, are needed to mitigate these dangerous conditions, using available evidence of potential poor outcomes associated with HV as justification to dismiss the implementation of therapeutic HV is debatable and remains a controversial subject among physicians. This review highlights various issues surrounding the use of HV as a means of controlling posttraumatic ICH, including indications for treatment, potential risks, and benefits, and a discussion of what techniques can be implemented to avoid adverse complications.

## Introduction

Physicians started to explore hyperventilation (HV) as a way to lower cerebral blood volume (CBV) during the 1920s (1). One of the earliest documented descriptions of this treatment dates back to 1959, when Lundberg reported the use of HV to reduce increased levels of intracranial pressure (ICP) (2). HV induces arteriolar vasoconstriction, which results in decreased cerebral blood flow (CBF) and, consequently, a decrease in CBV (3–5). As time went on, this easily implemented therapy was widely used for the management of intracranial hypertension (ICH) secondary to severe traumatic brain injury (sTBI) (6–8). By the mid-1990s, in neurosurgical centers located within the United States and the United Kingdom, the rate of HV utilization was 83 and 97%, respectively (6, 7). A European database analysis released in 2008 indicated that during the first 24 h after insult, physicians employed prophylactic HV in more than half of their TBI cases (9).

Although HV rapidly and effectively reduces ICP, the effects are transient and have not been associated with the improvement of final patient outcome (10, 11). Because HV can potentially trigger secondary cerebral ischemic lesions and create adverse repercussions that affect other organ systems, and the therapy remains a topic of controversy and vigorous debate (10, 11). Available evidence indicates that intense or prolonged prophylactic HV is detrimental and should be avoided, especially during the acute phase of sTBI; however, the therapy is still recommended as a way to temporarily manage life-threatening elevations of ICP (12). The goal of this review is to provide an update that evaluates the current studies describing HV for ICP control to determine if HV has a role in the management of acute brain injury.

## CO_2_ Physiology: Basic Concepts

Hyperventilation leads to an increase in alveolar ventilation (AV) (13, 14), the volume of air per minute that enters the respiratory zones (bronchioles, alveoli, etc.) that is also available for gas exchange (13). Because a portion of that volume remains in areas where gases do not diffuse into the bloodstream, AV can be determined by the following equation (13):

AV=RR (respiratory rate)×(VT [volume tidal]−VDS [volume dead space]).

Alveolar ventilation has an inverse relationship with the alveolar CO_2_ level; when AV increases, the alveolar CO_2_ levels decrease (13, 14). However, alveolar CO_2_ has a direct association with the partial pressure of arterial CO_2_ (PaCO_2_), which reflects the balance between the production and elimination of CO_2_ (13, 14):

$${\text{PaCO}}_{\text{2}}={\text{CO}}_{\text{2}}\text{ production}-{\text{CO}}_{\text{2}}\text{ elimination}.$$

A patient’s cellular production of CO_2_ is dependent on several variables, including diet, exercise, temperature, and hormone activity (thyroid) (13). CO_2_ production remains relatively stable and constant, except during severe hypermetabolic states. If CO_2_ transport and cardiac output remain unchanged, PaCO_2_ levels will be determined inversely by the rate of CO_2_ elimination through the AV (13).

**Figure 1:**
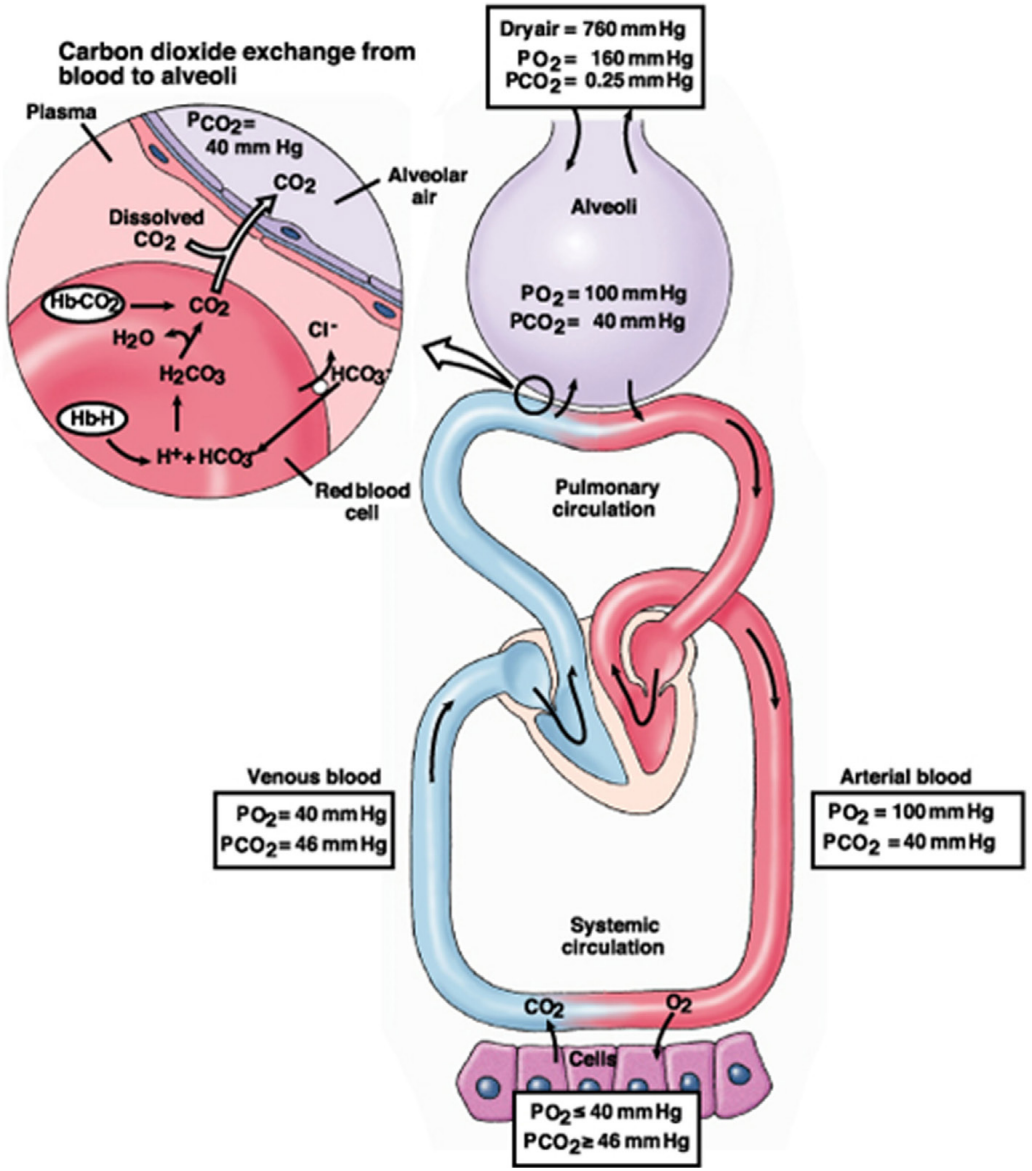
The CO_2_ physiology from cells to alveoli.

CO_2_ is a soluble and diffusible gas that is transported in three different ways: 10–15% of it is dissolved according to the PaCO_2_ (Henry’s Law); 20–30% of it is bound to plasma proteins and hemoglobin that form carbaminic complexes, and 65–70% of it is converted to bicarbonate/carbonic acid in the red blood cells and plasma (13). This third complex reaction helps maintain equilibrium between bicarbonate $\left({\text{HCO}}_{3}^{-}\right)$ and hydrogen (H^+^) ions (13, 14) (Figure 1).

Normal PaCO_2_ values fluctuate between 35 and 45 mmHg (4.7–6 kPa) at normal body temperature and at a sea level with barometric pressure of 760 mmHg (13, 14).

If body temperature decreases, the solubility of CO_2_ increases and PaCO_2_, consequently, decreases (13, 15). PaCO_2_ decreases 4.5% for each centigrade degree temperature decrease. The opposite occurs when temperature increases (Table 1) (15). At higher altitude, the barometric pressure decreases, stimulating AV, so normal PaCO_2_ levels are lower (Table 2) (16–20).

**Table 1:** Modification of normal PaCO_2_ values according to changes in central temperature ([ref]^15^).

Temperature (°C)	pH	PaCO_2_ (mmHg)
40	7.36	46.2
39	7.37	44.1
38	7.39	42
37	7.40	40
36	7.41	38.1
35	7.43	36.3
34	7.44	34.6
33	7.46	33
32	7.47	31.4
31	7.49	29.9
30	7.5	28.5

**Table 2:** The normal PaCO_2_ according to altitude and barometric pressure.

City (country)	Altitude above sea level (m)	Barometric pressure (mmHg)	Normal PaCO_2_ (mmHg)
Sea Level (13)	0	760	38.3
Colima (Mexico) (16)	494	717	37.1
Cordoba (Mexico) (16)	927	681.4	36
Orizaba (Mexico) (16)	1,248	656.1	35.2
Leon (Mexico) (16)	1,804	614.5	33.8
Puebla (Mexico) (16)	2,144	590.3	32.9
Mexico City (16)	2,238	583.8	32.7
Toluca (Mexico) (16)	2,651	556.1	31.7
Bogota (Colombia) (17)	2,640	560	31.2
Quito (Ecuador) (18)	2,850	543	31.6
Cusco (Peru) (19)	3,350	530	30.6
La Paz (Bolivia) (20)	3,577	496	30

## Changes in Cerebral Physiology During PaCO_2_ Reduction

The brain is one of the most metabolically active organs in the entire body. Because it lacks reserves of oxygen and glucose, two nutrients that are vital to maintaining vigorous physiochemical activity, it is important for the brain to have some system of continuous delivery through which these substrates can be received (21). This delivery system occurs through the CBF. CBF is so crucial that the brain has developed adaptive mechanisms to maintain adequate and constant flow despite changes in physiological variables or metabolic requirements (21–23). The maintenance of CBF is called “cerebral autoregulation,” and it is primarily achieved by the resistance arterioles (22, 23). By dilating or contracting in response to changes in arterial blood pressure, viscosity, gases, and metabolic demands, the arterioles can regulate CBF (22, 23).

## Cerebral Blood Flow

**Figure 2:**
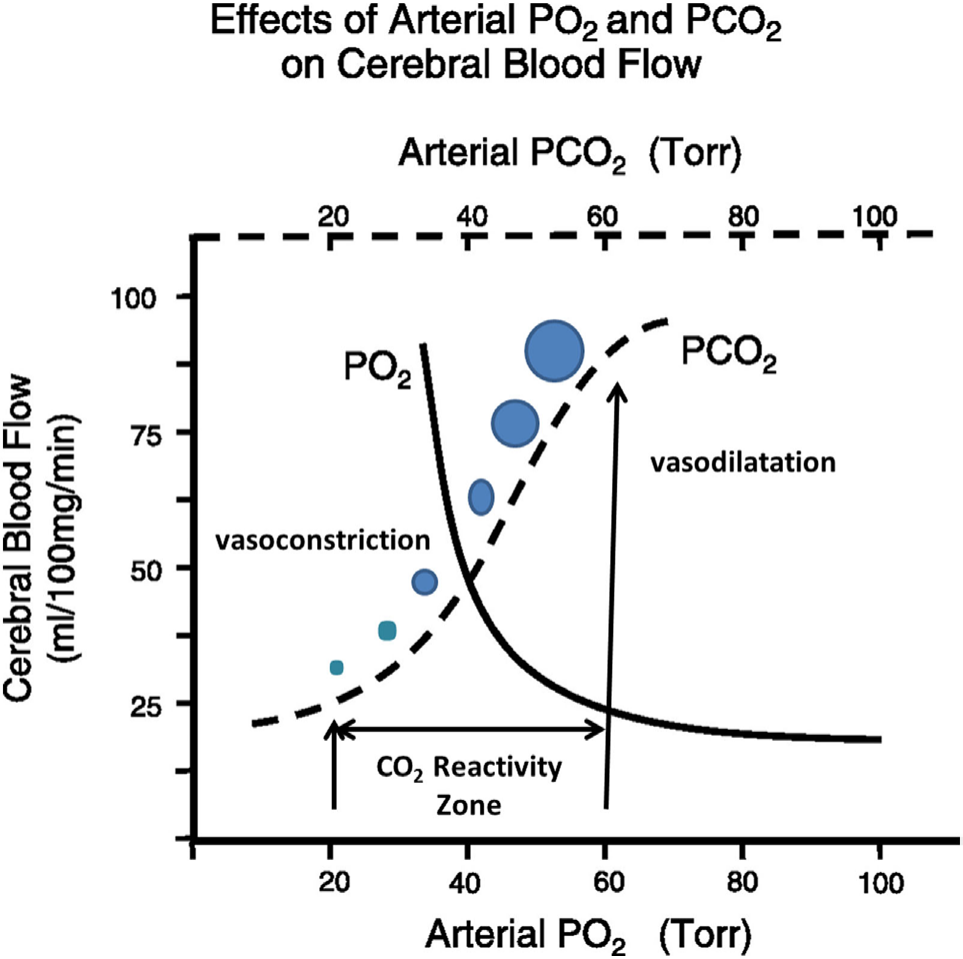
The effects of arterial CO_2_ on cerebral blood flow.

“CO_2_ reactivity” is the ability cerebral resistance arterioles possess to dilate or contract in response to changes in the partial pressure of arterial CO_2_. Dilation occurs when the partial pressure of arterial CO_2_ increases (PaCO_2_ > 44 mmHg indicates hypercapnia); the vessels contract if the PaCO_2_ level decreases (PaCO_2_ < 35 mmHg indicates hypocapnia) (3–5, 10, 11). However, this vascular activity only occurs within the 20–60 mmHg range of PaCO_2_ (24). The diameter of the vessels will not change if PaCO_2_ levels rise above or drop below that specific range. Because of these restrictions, when it is plotted on a graph, the autoregulatory curve depicting CBF according to shifting PaCO_2_ resembles a sigmoid function (Figure 2) (4, 25).

Changes in vessel diameter elicited by hypocapnia compared to those elicited by hypercapnia are not proportional (5). If the PaCO_2_ increases to 80 mmHg, vasodilation will increase CBF by 100–200%, causing a release of catecholamines and an increase of metabolic activity. On the other hand, for every millimeter of mercury that the PaCO_2_ decreases, the CBF will decrease by 3%; thus, PaCO_2_ levels between 20 and 25 mmHg are associated with a CBF reduction of 40–50% (5, 24).

The pial arterioles responsible for these instances of dilation and contraction are less than 50 µm in diameter. The vascular endothelium reacts to changes in pH that occur within the perivascular space by releasing mediators that regulate the endothelium and vascular smooth muscle (5, 26). These vasoactive factors are thought to include nitric oxide, prostaglandins, cyclic nucleotides, potassium, and calcium (5).

Cerebral blood flow is not normally homogeneous; it varies according to the metabolic rate and activity of each region (27). Cerebrovascular reactivity to CO_2_ can also vary, depending on location or circumstance (28). During sTBI, especially during the first few hours, CO_2_ reactivity is exacerbated, especially in areas that are adjacent to contusions or subdural hematomas (29–32). For these reasons, changes in normal levels of CO_2_ are potentially dangerous secondary insults that can drastically impact brain physiology (11).

## Cerebral Blood Volume

In adult humans, normal CBV is 3–4 ml per 100 g of parenchymal tissue (5). Although changes in the diameter of the cerebral blood vessels might alter the total CBV, 70% of the total blood volume contained in the brain corresponds to the venous system (33, 34). Because veins and capillaries do not react to fluctuations in PaCO_2_, any changes in the CBV following incidents of hypercapnia or hypocapnia can be attributed only to changes in the arterial blood volume (33, 34). It has been estimated that HV reduces CBV by approximately 0.049 ml/100 g per millimeter of mercury CO_2_ reduction (5). If only 30% of the total CBV is located in the arteries and only pial vessels respond to changes in PaCO_2_, a hypocapnia-induced CBF decrease of 30% will only result in a CBV decrease of 7% (35). In this manner, a pronounced decrease in CO_2_ can create a substantial decrease in CBF, but has little effect on the corresponding CBV and ICP (35). It has been suggested that the CBV response to hypocapnia is further diminished during arterial hypotension, specifically when the mean arterial blood pressure (MABP) range is reduced from 154 to 114 Hg (5).

## Intracranial Pressure

In accordance with the Monro–Kellie hypothesis, alterations in the CBV will create ICP changes only after spatial compensatory mechanisms are exhausted (10, 11, 14). These compensatory mechanisms include changes in cerebrospinal fluid (CSF) and blood volume principally through increased venous return to the heart and deviation of CSF to the spinal channel. Hypercapnia triggers vasodilation, which leads to an increase in CBV and a subsequent increase in ICP; hypocapnia triggers vasoconstriction, which leads to a decrease in CBV and a subsequent decrease in ICP (10, 11, 14). HV is a therapy that uses the conditions of hypocapnia to trigger vasoconstriction within the resistance arterioles in the cerebral parenchyma in order to reduce ICP. Doing this modifies the absolute value and morphology of the ICP pulse wave by decreasing the P2 (tidal wave) component (36).

## Brain Metabolism

Hypocapnia increases cerebral metabolic activity through various mechanisms. It induces the release of excitatory amino acids (*N*-Methyl-d-aspartate and glutamate) and increases neuronal excitability, glucose consumption, and the metabolic rate of O_2_ (CMRO_2_) (11, 14). It also potentiates and prolongs convulsive activity (11, 14).

## Cerebral Oxygenation

Hypoxia occurs when the body or a specific region of the body does not receive or is unable to process an adequate amount of oxygen to meet its metabolic demands (37–39). Tissular hypoxia can be local or global, but both variants can be detected using bedside cerebral monitoring that measures either the tissular pressure of O_2_ (ptiO_2_) or the venous saturation of O_2_ in the bulb of the jugular vein (SvjO_2_) (40). There are four different pathways through which hypocapnia can cause or contribute to tissular hypoxia (37–39):

- Vasoconstriction brought upon by hypocapnia can cause a reduction in CBF, resulting in “ischemic hypoxia” (3, 5, 10, 11, 14).
- A reduction in carbon dioxide levels can impair gas exchange in the lungs, triggering “hypoxemic hypoxia” (10, 11).
- The oxygen–hemoglobin (Hgb) dissociation curve can shift to the left as a result of hypocapnia, which increases the Hgb’s affinity for O_2_ and hinders the release of O_2_ into the cells which is also known as “high affinity hypoxia” (10, 11).
- Heightened neuronal excitability and cerebral metabolism brought upon by hypocapnia increases metabolic needs, resulting in hypoxia (11, 14).

**Figure 3:**
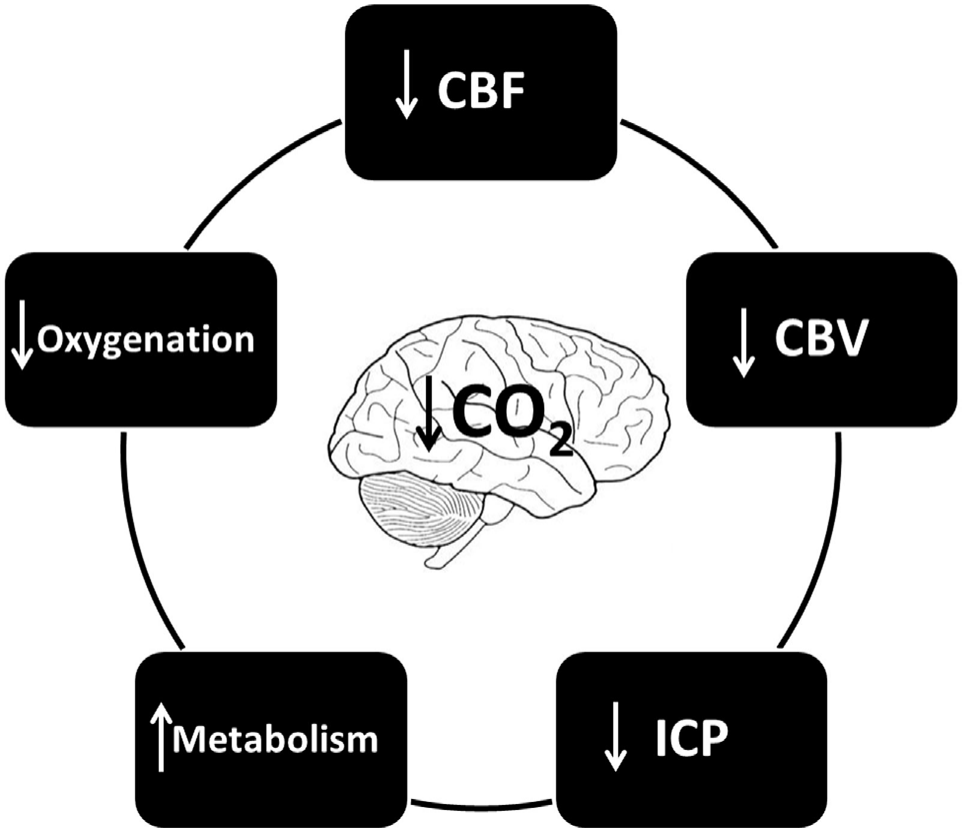
The cerebral effects of hypocapnia.

The various changes that hypocapnia induces with regard to cerebral physiology are depicted in Figure 3.

## The Systemic Effects of Hypocapnia

Induced hypocapnia affects all organ systems (10, 11, 14). When evaluating hypocapnia for the purposes of potentially administering therapeutic HV, one should also take into account the effects of mechanical ventilation (10, 11, 41, 42). Because patients suffering from sTBI might concomitantly present contused lesions in the lung parenchyma, microaspirations of gastric content, or acute respiratory distress syndrome (ARDS), they may require protective ventilation with low VT and high levels of positive end-expiratory pressure (PEEP) (10, 11, 41, 42). Therapeutic HV is achieved by increasing the RR or VT. Increasing the VT can be detrimental because of the stress it places on the body; it induces alveolar stretching, which causes cytokine release and inflammation both locally and systemically (10, 11, 41–43).

Hypocapnia decreases blood perfusion to renal tissue, gastrointestinal tissue, and skin and skeletal muscle tissue; it also provokes an increase in adhesion and platelet aggregation (10, 11, 14). Low PaCO_2_ levels incite bronchoconstriction, attenuate hypoxic pulmonary vasoconstriction, inhibit the production of surfactant, and increase the permeability of the alveolo-capillary membrane and upper airways (11, 14). Several variables can compromise gas exchange and increase a patient’s susceptibility to infections. These include: atelectasis, edema, reduced compliance, pulmonary defense mechanism alterations, ventilation–perfusion ratio alterations, and changes to the shunt fraction (11, 14). Respiratory alkalosis (a disturbance in the acid/base balance associated with decreased levels of potassium, calcium, and phosphate in plasma) complicates tissue oxygenation by shifting the O_2_/Hgb dissociation curve to the left (10, 11).

Hypocapnia-induced vasoconstriction compromises coronary blood flow and increases the risk of coronary spasm. It also increases myocardial metabolic demands, which may increase a patient’s risk for myocardial ischemia. This is especially true if the patient has predisposing factors or a history of heart disease (10, 11, 14). Hypocapnia also promotes reperfusion injury and encourages the development of arrhythmias, specifically, atrial fibrillation (11).

## Hypocapnia and Neurotoxicity

Low levels of PaCO_2_ produce neurotoxic effects by inducing the release of cytotoxic excitatory amino acids, increasing dopamine levels in the basal ganglia, and promoting the incorporation of choline into the phospholipids of cell membranes (44–46).

## Hyperventilation and Timing Restrictions

Cerebral arteriolar reactivity to CO_2_ is dependent on perivascular pH changes (26). HV induces hypocapnic alkalosis, which rapidly triggers buffer mechanisms that attempt to normalize changes made to extracellular space and (CSF) levels (10, 11). During this time, there is a rapid cellular efflux of hydrogen ions (H^+^) that bind to bicarbonate $\left({\text{HCO}}_{3}^{-}\right)$ and generate carbonic acid (H_2_CO_3_), which dissociates in water (H_2_O) and CO_2_ (10, 11). At the same time, extracellular bicarbonate is exchanged with chloride from the intracellular space (10, 11). These buffer mechanisms are inefficient because they rapidly deplete; if hypocapnia persists, alkalosis will perpetuate.

A slower but more efficient buffer occurs at the proximal renal tubular level, where ${\text{HCO}}_{3}^{-}$ reabsorption is inhibited at the same time H^+^ secretion is stimulated (10, 11). These reactions begin minutes after hypocapnic alkalosis originates and are maintained for hours or even days, allowing the CSF and perivascular pH to normalize 6 h after hypocapnia begins; HV naturally becomes less effective after this buffer pathway becomes activated (10, 11). Clinical studies have demonstrated a 40% decrease in CBF when PaCO_2_ levels are 20 mmHg; but, after 4 h of HV, CBF begins to normalize (47). CO_2_ levels after HV therapy also requires time to normalize; if PaCO_2_ rapidly increases, the perivascular pH (normalized by buffer systems) will decrease, causing local acidosis, vasodilatation, and an increase in CBV and ICP (also known as “rebound hyperemia”) (11, 48).

## Clinical Evidence of Hyperventilation Therapy in the Management of sTBI

For many years, HV was a key therapy in the control and prevention of posttraumatic ICH (6–8, 49–51). Oertel et al. reported that HV is a potent and more effective tool to lower elevated ICP levels when compared to increase mean arterial pressure (MABP) or decreased brain metabolism with propofol (52). Multiple studies indicate that the mechanism by which HV decreases ICP is vasoconstriction and CBF reduction (3–5, 10, 11). As CBF decreases, the risk of ischemia is a latent danger (8, 10–12). This is a controversial matter that is subject to much debate.

Immediately following trauma, CBF decreases to about 40% and the CMRO_2_ possibly decreases as well (53–56). After at least 48 h, this period is followed by two consecutive phases of “relative hyperemia” (in which CBF increases above metabolic demands) and vasospasm (57). The posttraumatic brain is extremely susceptible to ischemic damage (58–61). In almost half of all reported sTBI cases, the autoregulatory pressure mechanism is compromised; therefore, CBF becomes “pressure dependent” (58–61). Under different circumstances, the autoregulation pressure curve might shift to the right, which will increase the cerebral perfusion pressure (CPP) limit to help prevent ischemia (58–61). Because the brain needs to achieve a higher CPP during the acute phase of sTBI, it is highly recommended that hypotension should be avoided during this period.

**Figure 4:**
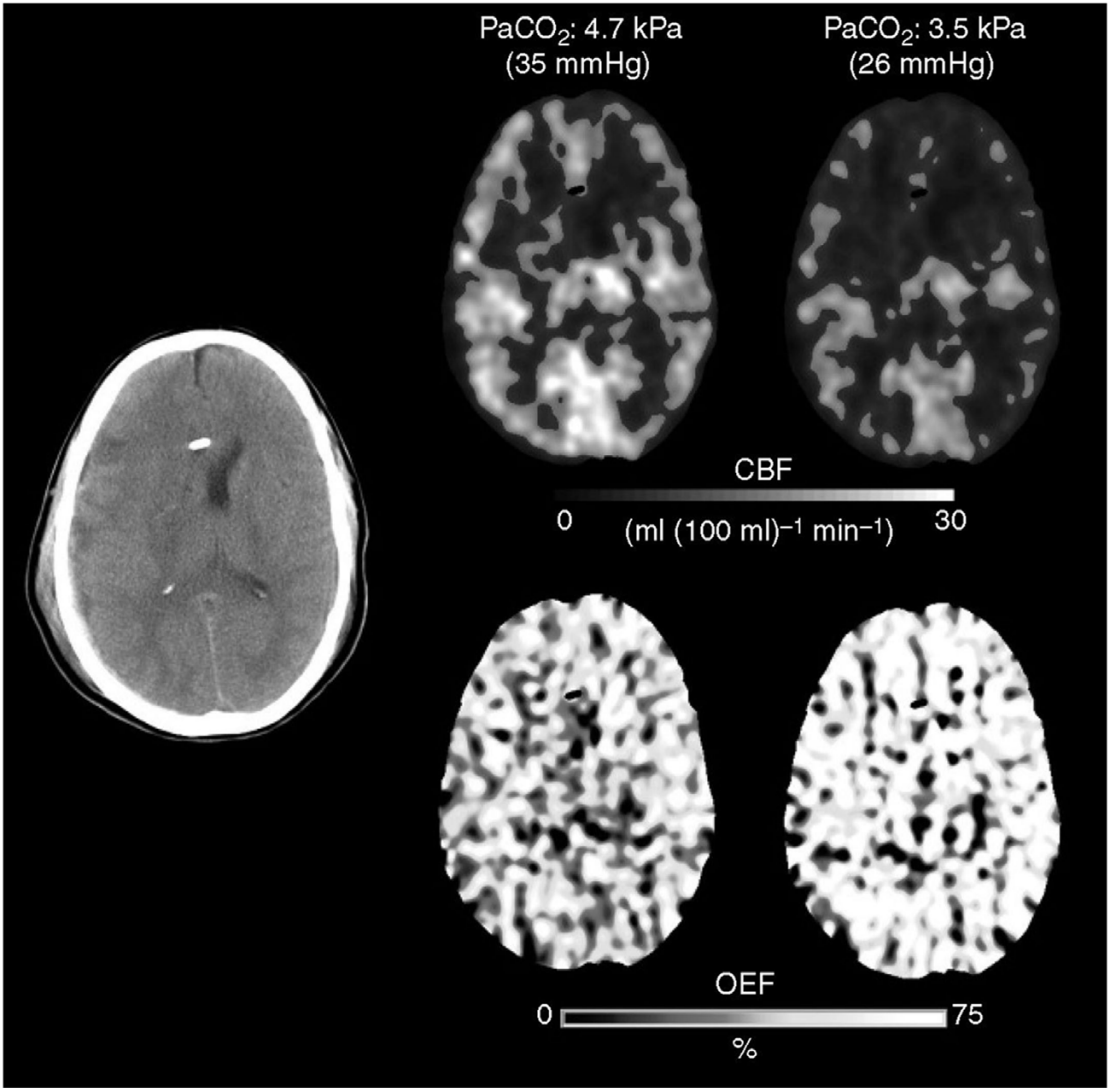
The Xenon CT imaging that showed cerebral blood flow (CBF) decrease and O_2_ extraction fraction (OEF) increase after hyperventilation.

Much like CBF, arteriolar vasoreactivity to CO_2_ can vary according to region. CO_2_ reactivity is habitually maintained and exacerbated during the initial phase of trauma, especially in areas adjacent to contusions or subdural hematomas; a close proximity to these areas increases the likelihood of ischemia occurring in those regions (Figure 4) (31, 33, 62). If CO_2_ reactivity becomes compromised, it is generally by a terminal event associated with poor results (53, 62–64). For these reasons, it is a key point at this stage in treatment to maintain CBF within normal limits to provide adequate CPP (CPP = MABP − ICP) and blood viscosity while avoiding resistance vessel constriction (12, 58–61).

## Hyperventilation and Cerebral Ischemia

Davis was the first to observe the slowing of electroencephalogram waves after HV and attributed this behavior to ischemia (65). Cold evaluated regional CBF (rCBF) using a Xenon (Xe-CT) technique before and after HV between the first day of admittance and 3 weeks post-sTBI (66). Under these conditions, mean ICP was reported to be 19 mmHg and PaCO_2_ levels changed from 36 to 26 mmHg. The hyperventilated group demonstrated three times as many oligemic regions (defined by a CBF < 20 ml/100 g/min), and their areas of severe CBF reduction (<15 ml/100 g/m) increased from 0.1 to 3%. These observations were more evident in cerebral hemispheres with lower basal CBF. There was also a strong correlation between HV, reductions in rCBF, and poor patient outcome (66).

Multiple clinical studies examining sTBI have confirmed that HV causes significant reductions in CBF (67–69). But observing a reduction in CBF is not enough to accurately diagnose ischemia. To ascertain a valid diagnosis, tissue hypoxia must be associated with any observed decrease in CBF (37–40). Although they are not available in real time at the bedside, it is still important to perform metabolic studies, such as positron emission tomography (PET) (70).

A group from Cambridge conducted a series of studies in which they analyzed the effects of HV in patients with sTBI using PET (71–73). A common denominator among the patients was the absence of ICH (71–73). In the group’s first study, 33 patients were tested within the first week of the trauma, and four were evaluated within the first 24 h of admission (71). The researchers decreased PaCO_2_ levels from a baseline of 36–29 mmHg. HV elevated CPP and decreased ICP and CBF, which increased the number and volume of areas that reached the hypoperfusion range. However, these changes were not associated with global ischemia since SvjO_2_ and the arterial–venous difference of O_2_ (AVDO_2_) remained within normal limits (71).

In a subsequent study, 13 patients were analyzed during the same posttrauma period to test the hypothesis that diffusion alterations in microcirculation contribute to tissue hypoxia (72). The patients were monitored with PET, O_2_ extraction fraction (OEF), ptiO_2_, and pvO_2_ (72). Again, after inducing HV at a 29 mmHg of PaCO_2_, CBF decreased. Areas in the range of hypoperfusion and hypoxia (ptiO_2_ < 10 mmHg) showed less reserve capacity to extract O_2_, which increased the risk of ischemic damage in these regions (72).

Using a modified PET technique [O_2_ (15)], Coles et al. evaluated CBF, CMRO_2_, CBV, and OEF in 30 sTBI patients within 10 days of trauma (73). Hypocapnia (PaCO_2_ = 29 mmHg) caused a decrease in CBF, an increase in the volume of ischemic areas, and an increase in OEF. CMRO_2_ increased, but the response was heterogeneous. Twenty-eight percent of hyperventilated individuals showed a marked decrease in CMRO_2_. CMRO_2_ correlated with neurophysiological monitoring findings (73).

In another series of sTBI patients with normal ICP values, the effects of HV and mannitol on CBF and metabolic variables were measured using a Doppler ultrasound device and blood samples were taken from a jugular catheter (74). The timing of the study was not specified. The authors reported that, when compared to the 20% (0.5 g/kg) mannitol group, the group hyperventilated at a target PaCO_2_ of 32 mmHg had a lower CBF and CMRO_2_; glucose utilization (CMRGlu) and lactate production (CMRL) also increased in this group, which was interpreted by the authors as indicative of anaerobic hyperglycolysis (74).

Using Xe-CT, Stringer analyzed rCBF in 12 individuals with various etiologies, four of which were TBI patients (75). HV was induced heterogeneously with varying expired CO_2_ values (ETCO_2_), three of them being lower than 20 mmHg. The study showed a decrease in rCBF. No metabolic parameters were measured (75).

Using thermodiffusion and microdialysis, Marion analyzed rCBF and tissue hypoxia markers in the extracellular fluid of 20 individuals with sTBI before and after HV at a target of 24.6 mmHg (76). Patients maintained normal ICP values. The authors analyzed “apparently healthy areas” adjacent to contusions or subdural hematomas during two-time intervals: 24–36 h and 3–4 days posttrauma (76). After HV, an increase in glutamate, lactate, and the lactate/pyruvate relationship (L/P) was observed to be 10% over basal values. CBF decreased by the same proportion. These changes were seen in both time intervals, but they were more frequently observed during the early stage post-TBI (76).

Using PET, Diringer tested the behavior of CBF, CBV, CMRO_2_, OEF, and CvO_2_ after HV under a pre-specified target PaCO_2_ of 30 mmHg. Patients were analyzed an average of 11 h after sTBI. Of the nine individuals studied, only five had an ICP greater than 20 mmHg (77). Reductions in CBV, CBF, and CvO_2_ were observed. There was no apparent ischemia or energy dysfunction since CMRO_2_ remained unchanged at the expense of an increase in OEF (77). Two years later, the same group used the same methodology to compare the effects of HV on patients with, and without ICH on posttrauma days 1 and 5 (78). The results they obtained and the conclusions they reached were no different from those reported in the previous study (78).

## Hyperventilation and Cerebral Oxygenation

PaCO_2_ affects the measurements taken by both global (SvjO_2_) and regional (ptiO_2_) oxygenation monitoring methods. HV reduces ICP levels, and clinical studies have demonstrated a simultaneous decrease in SvjO_2_ values (79–81). When analyzing the impact that ETCO_2_ levels have on CBF and ptiO_2_, a direct relationship has been observed between these variables, especially in the range of ETCO_2_ from 20 to 60 mmHg (82). When HV is more intense and ETCO_2_ levels are lower, the likelihood of detecting tissue hypoxia using ptiO_2_ monitoring increases (83). Hypocapnia is one of the secondary insults that is likely to trigger tissue hypoxia (84). The effect HV has on tissue oxygenation becomes more dramatic as time goes on; it has the most impact around 5 days posttrauma (85, 86). This phenomenon is associated with poor results (86–88). Multiple clinical studies have established the deleterious effect HV has on ptiO_2_ levels. In sTBI, a lower ptiO_2_ has clearly become an independent predictor of mortality and poor patient outcome (86–89).

## Hyperventilation and sTBI Outcome

Only a few studies have established a correlation between patient outcome and HV. Two small studies concluded that mortality and poor functional outcomes were associated with HV when increasing the volume of areas with low CBF into the ischemic range (66) or when ptiO_2_ levels decreasing along with increasing CO_2_ reactivity 5 days after trauma (86).

Gordon reported a large retrospective series of patients treated with prolonged hyperventilation (90). 251 patients with sTBI were studied, 51 of whom were hyperventilated (PaCO_2_ between 25 and 30 mmHg). The time period of HV varied between 6 h and 41 days (mean 10 days). The HV group had a lower mortality (9.8 vs. 32.8%); however, the number of survivors with severe neurological sequela notably increased. Patients who experienced a complete recovery did not differ between groups (90). The authors of the paper gave few details about their methodology; their reported clinical data was also incomplete.

There is only one prospective, controlled, and randomized study that evaluated the final outcome of sTBI patients who were treated with deep and prolonged (5 days) HV (91). Three groups were evaluated in this study: patients who received normoventilation (PaCO_2_ 35 mmHg), patients who received HV at a PaCO_2_ of 25 mmHg, and patients who received HV and THAM (tromethamine). The THAM acted like a buffer, preventing pH changes within the extracellular cerebral fluid and CSF in order to extend the period during which HV was effective. Prior to randomization, patients were stratified into two groups according to the motor component of the Glasgow Coma Scale (GCS): ≤3 or >3 points. Favorable results at 3 and 6 months of the event were significantly lower in the HV group, especially for patients who had a better clinical status at the time of admission (motor GCS 4–6). After a year had passed from the date of trauma, the differences between the groups were no longer significant. When evaluating CBF (Xe133) and AVDO_2_, there was no evidence of ischemia in any of the three groups (91).

The study’s conclusions should be interpreted with caution. In the first place, the clinical and tomographic characteristics of the patients were not well balanced between the groups. The number of patients per group was also small, so there could have been statistical errors of type α (false positives). The control group was also hyperventilated with a mean PaCO_2_ at 31 mmHg. Third, it is apparent that HV was used prophylactically because only 14% of the individuals in the control and HV groups and 5% of the HV and THAM group had high ICP values. Finally, when analyzing the outcome at 12 months posttrauma, the best results correspond to HV + THAM group.

## Target PaCO_2_ in the Management of sTBI

The Cochrane collaboration concludes that there is an insufficient amount of evidence to clearly establish whether hyperventilation therapy in the management of sTBI is beneficial or detrimental (92). In emergent medical situations, Brain Trauma Foundation (BTF) guidelines recommend a brief period of hyperventilation (HV) (15–30 min to target PaCO_2_ 30–35 mmHg) to treat acute neurological deterioration reflecting increased ICP (12). However, in patients with TBI, the targeted PaCO_2_ of normoventilation is 35–40 mmHg with a pulse oximetry of 95% or greater and/or PaO_2_ of 80 mmHg or greater (12). If the patient is refractory to all other treatments, including hypertonic saline, sedation, and paralytics, a prolonged period of HV with brain oxygenation monitoring may be required to relieve ICH (12).

## Rules to Take into Account before Hyperventilating a Patient with sTBI

**Figure 5:**
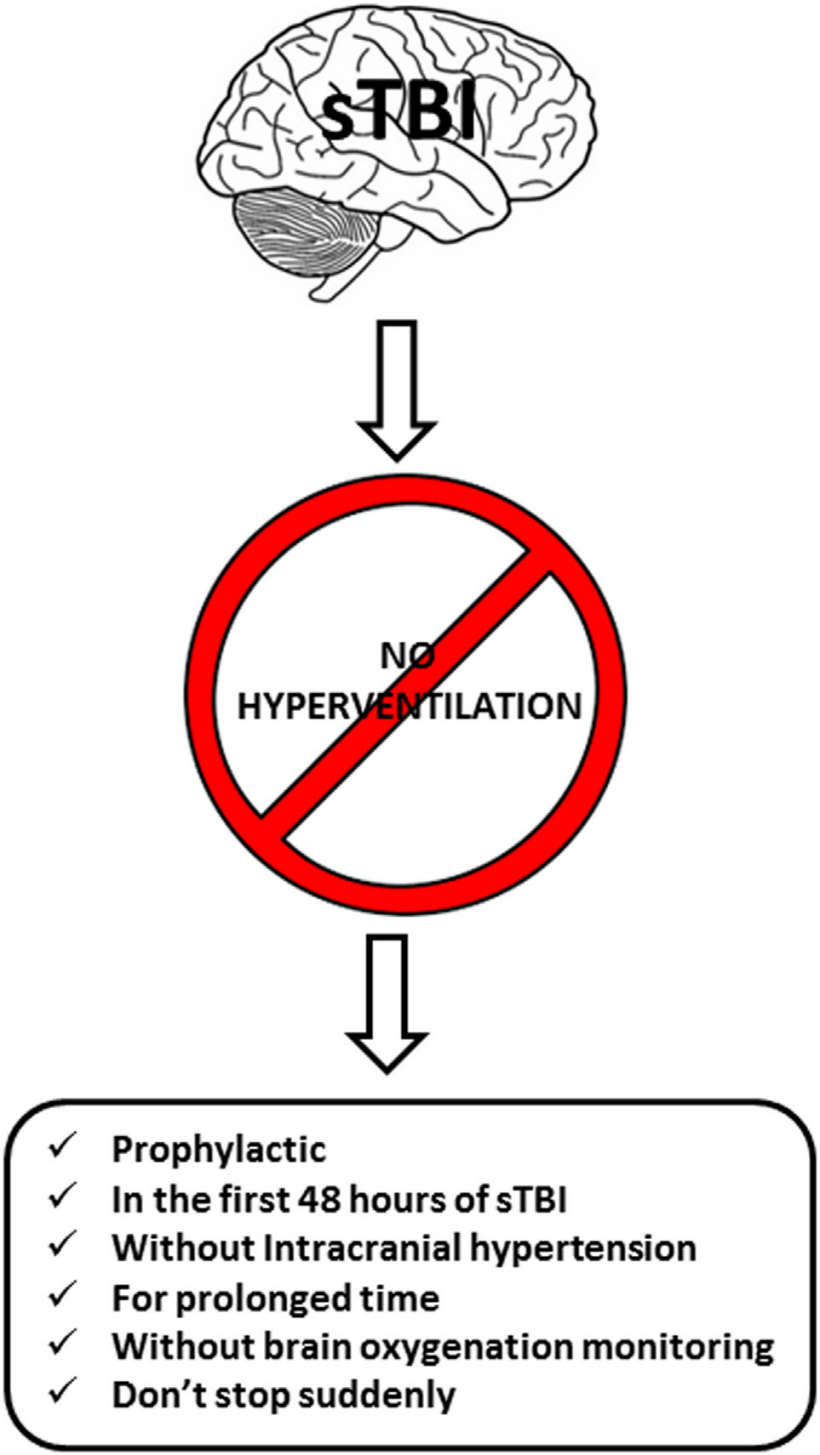
Six DO NOT rules.

Hyperventilation has a place in the management of ICP. For physicians to determine if there is a correct and sufficient indication for treatment with minimal possible risk for the patient, a systematic approach based on current scientific evidence must be undertaken. The authors recommend the following guidelines Figure 5:

- DO NOT hyperventilate prophylactically. HV will not prevent ICP increase, nor will it improve the final outcome.
- DO NOT hyperventilate in the absence of ICH.
- DO NOT hyperventilate for prolonged periods of time. At 4–6 h, buffer systems normalize the pH of the perivascular space, thereby negating the effects of hypocapnia on the cerebral vasculature.
- DO NOT hyperventilate within the first 24 h of sTBI, when the risk of ischemia is greatest.
- DO NOT hyperventilate without oxygenation monitoring. Consider using transcranial Doppler and measuring CO_2_ levels through the determination of expired ETCO_2_ levels or arterial gases. Because ischemic hypoxia is a latent and dangerous risk, monitor cerebral oxygenation globally (SvjO_2_), locally (ptiO_2_), or both despite the low level of evidence of this recommendation.
- DO NOT suddenly stop HV. Abrupt cessation will increase the risk of ICP elevation rebound.

## Ideal Conditions for Hyperventilation

The cerebral autoregulatory mechanism is compromised in more than half of reported sTBI cases, but HV can help ameliorate those circumstances (93, 94). However, the benefits derived from HV are transient and achievable only through moderate HV. A Doppler study examining 10 patients with sTBI demonstrated that HV at a PaCO_2_ of 28 mmHg significantly improved cerebral autoregulation, but the benefit was lost when HV was reduced to a PaCO_2_ of 23 mmHg (95). Another group composed of 30 sTBI individuals with normal ICP values evidenced improvement in autoregulation when PaCO_2_ levels were temporarily decreased from 38 to 33 mmHg (96).

Hyperventilation is recommended as a temporary measure to reduce high levels of ICP in the following situations:

➢ Herniation syndromes. These are syndromes involving the deterioration of neurological status (mydriasis, abnormal motor postures) secondary to expansive lesions (epidural, subdural hematomas, etc.) as a bridge to surgical resolution (12).
➢ Life-threatening elevations of ICP. For example, type A plateau waves, while investigating triggers and expecting the effect of osmotherapy (12).
➢ Refractory ICH. HV is used in conjunction with second level measures, such as decompressive craniectomy, hypothermia, or high doses of barbiturates (12).
➢ ICH secondary to “hyperemia.”

In approximately 20% of sTBI cases, ICP elevation correlates with high CBF in excess to metabolic demands, mainly in young individuals (53, 97, 98). In these situations, based on the principle of coupling between CBF and CMRO_2_, the concept of “optimizing HV” emerges in order to lower CBF and, consequently, ICP without modifying CMRO_2_ (99–102). By implementing this approach alongside cerebral oxygenation monitoring through SvjO_2_ and associated variables, Cruz et al. calibrate HV to a target PaCO_2_ that varied from 18 to 30 mmHg. As a result, ICP decreased without modification in CMRO_2_ while OEF increased (98–102). In another series of sTBI patients, it was reported that HV contributed to the stabilization and improvement of brain glucose uptake (101).

Hyperventilation optimization has its limitations. It is based on global monitoring that does not take into account the regional differences in CBF, metabolism, or CO_2_ reactivity. ICP compartmentalization and lesion type (focal or diffuse) are similarly disregarded in this manner. There does not exist a consistent definition of “hyperemia,” especially about the monitoring methods available at the bedside. There are also multiple limitations to the method of oxygenation monitoring itself, which can make it difficult to interpret the data obtained from said monitoring.

## Hyperventilation Techniques

**Figure 6:**
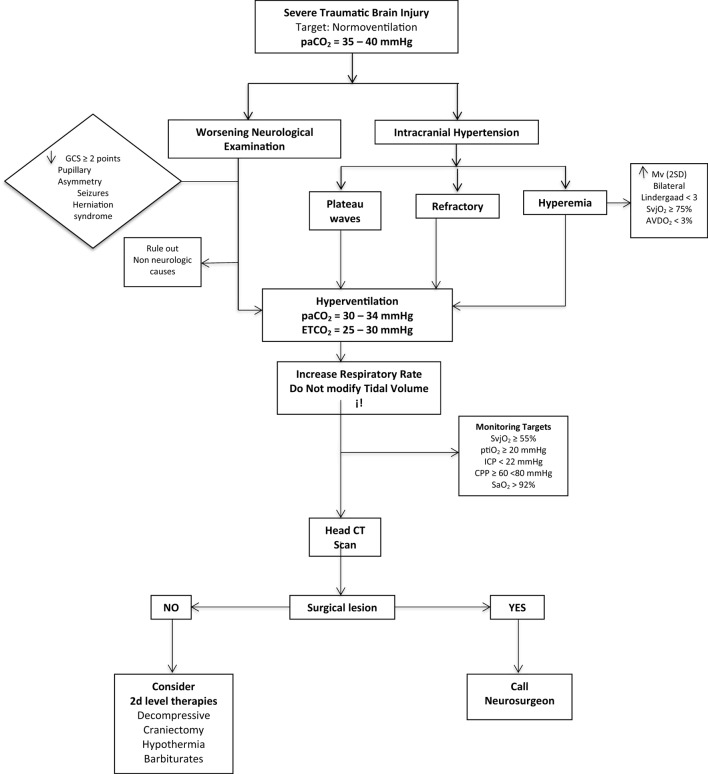
The practical algorithm to perform HV safety. Hyperemia definition: increase in mean velocity (mv) of mean cerebral artery in TCD (TRANSCRANIAL DOPPLER) of more than 2 standard deviations.

In Figure 6, we outline a practical algorithm summarizing the concepts one should take into consideration when hyperventilating a patient.

## Conclusion

Patients with an elevated ICP require emergent intervention to prevent deleterious consequences. Under certain conditions, when cerebrovascular CO_2_ reactivity is intact, HV can be used temporarily to induce hypocapnia to elicit arteriolar vasoconstriction with the aim of decreasing CBF and, ultimately, ICP. The cerebral effects of hypocapnia are transient. Because profound and prolonged HV carries the risk of ischemia, it is important that the therapy be closely monitored to prevent any adverse cerebral effects.

Hyperventilation has serious systemic consequences. It should not be implemented during the first 24 h of trauma when CBF is markedly reduced. Prophylactic HV or HV without an indication of elevated ICP will not yield any benefits. Current recommendations suggest that a brief period of HV (15–30 min) with a PaCO_2_ target of 30–35 mmHg and CPP target of 60–70 mmHg coupled with close oxygenation neuromonitoring is an effective method of controlling ICH during the acute-phase of sTBI.

## Author Contributions

All authors contribute to the preparation of the manuscript in the same manner.

## Conflict of Interest Statement

The authors declare that the research was conducted in the absence of any commercial or financial relationships that could be construed as a potential conflict of interest. The reviewer, BA, and handling editor declared their shared affiliation, and the handling editor states that the process nevertheless met the standards of a fair and objective review.
